# Exciton Diffusion in Organic Nanofibers: A Monte Carlo Study on the Effects of Temperature and Dimensionality

**DOI:** 10.1038/s41598-018-32232-5

**Published:** 2018-09-19

**Authors:** Leonardo Evaristo de Sousa, Demétrio Antônio da Silva Filho, Rafael Timóteo de Sousa, Pedro Henrique de Oliveira Neto

**Affiliations:** 1 Institute of Physics, University of Brasília, 70.919-970 Brasília, Brazil; 2 Department of Electrical Engineering, University of Brasília, 70.919-970 Brasília, Brazil

## Abstract

Organic nanofibers have found various applications in optoelectronic devices. In such devices, exciton diffusion is a major aspect concerning their efficiency. In the case of singlet excitons, Förster transfer is the mechanism responsible for this process. Temperature and morphology are factors known to influence exciton diffusion but are not explicitly considered in the expressions for the Förster rate. In this work, we employ a Kinetic Monte Carlo (KMC) model to investigate singlet exciton diffusion in para-hexaphenyl (P6P) and *α*-sexithiophene (6T) nanofibers. Building from previous experimental and theoretical studies that managed to obtain temperature dependent values for Förster radii, exciton average lifetimes and intermolecular distances, our model is able to indicate how these parameters translate into diffusion coefficients and diffusion lengths. Our results indicate that these features strongly depend on the coordination number in the material. Furthermore, we show how all these features influence the emitted light color in systems composed of alternating layers of P6P and 6T. Finally, we present evidence that the distribution of exciton displacements may result in overestimation of diffusion lengths in experimental setups.

## Introduction

Exciton diffusion is one of the key processes behind the operation of organic optoelectronic devices^1–3^. In organic photovoltaics, for instance, diffusion is responsible for the arrival of excitons at the interface where charge separation may occur^4,5^. Thus, a long diffusion length may translate into highly performant devices. For this reason, a great interest has been shown in predicting and measuring exciton diffusion length in organic materials^6,7^.

Among the materials employed in optoelectronic applications, organic nanofibers have received considerable attention thanks to their capacity to self-assemble and the tunability of their optical properties^8–10^. In particular, para-hexaphenyl (P6P) has been found to crystallize in nanofiber shape with special ease^11,12^. Furthermore, it has been shown that deposition of *α*-sexithiophene molecules on P6P nanofibers also results in highly crystalline structures^13^. These findings make these two molecular species interesting material in which to investigate exciton diffusion.

The mechanism responsible for singlet exciton diffusion is the Förster resonance energy transfer^14–16^. This is a non-radiative transition that requires the existence of an overlap between the emission and absorption spectra of the donor and acceptor molecules, respectively. The rate of this transfer is given by^16,17^

1 $${k}_{F}=\frac{1}{{\tau }_{D}}{(\frac{{R}_{F}}{r})}^{6}$$

where *τ*_*D*_ is the radiative lifetime of the donor species, *r* is the intermolecular distance and *R*_*F*_ is the Förster radius, a characteristic distance for which the Förster transfer rate equals the emission rate of the donor molecule. The above expression is known to overestimate transfer rates when intermolecular distances are similar or inferior to the corresponding transition dipole^18^. In the 1 nm range, which is the minimum intermolecular distance we consider here, these issues are mitigated.

When decay pathways other than exciton recombination are neglected, the probability of Förster transfer may be written as

2 $${\rm{\Phi }}(r)=\frac{1}{1+{(\frac{r}{{R}_{F}})}^{6}}$$

In spite of the simplicity of the above expressions, different features that affect exciton diffusion are not explicitly taken into account. Two such features that are known to play an important role in this process are temperature and morphology^19–22^. To take into account these more complex issues, Kinetic Monte Carlo (KMC) models are often employed in the study of exciton diffusion^7,18,23–26^. These models must be able to calculate Förster rates and probabilities to decide the behavior of each exciton at every step of the simulation. Exciton lifetimes, displacements and speeds are then registered allowing for the calculation of several properties. An analytical approach is also possible in some cases. Diffusion coefficients may be estimated by ${R}_{F}^{6}/\tau {r}^{4}$ for the one dimensional case with equal spacing between sites. In the two dimensional case, however, the distances between a site and all its neighbors are not equal. This affects the transfer rates, the recombination probability and ultimately the diffusion length. Furthermore, analytical models are impractical when studying systems composed by more than one material, since the Förster radii are different, resulting in an asymmetry between the possibility of Förster transfers from one material to the other.

When it comes to temperature effects, our previous work that combined experimental results, a KMC model and a genetic algorithm has shown that the average Förster radius in P6P presents a temperature dependence in the 80 K to 300 K temperature range that can be modeled by the following expression^27^:

3 $${R}_{F}(T)={R}_{0}+{R}_{1}\exp \,(-\frac{{E}_{A}}{kT}),$$

with *R*_0_ = 13.8 Å, *R*_1_ = 15.8 Å and *E*_*A*_ = 14.8 meV. The methodology relied upon experimental time-dependent photoluminescence spectra of P6P to obtain this result. This expression may be interpreted as taking into account two aspects of exciton diffusion: first, an initial downhill migration process that is temperature independent and dominant at low temperatures. In this process, excitons hop towards lower energy sites where they become trapped; second, this process is then combined with a temperature activated behavior that dominates at higher temperatures, resulting in larger exciton diffusion lengths. Such a behavior has been observed for singlet exciton diffusion in polymers, for instance^6^.

On the other hand, the same study has also shown that exciton diffusion in 6 T is mainly temperature independent: for an average intermolecular distance of 10 Å, the Förster radius in 6 T can be estimated to be 24 Å for any temperature in the investigated range. This is in agreement with experimental results that found exciton diffusion lengths do not change with temperature^28^. This suggests very low energetic disorder in 6 T, which would prevent the appearance of a temperature activated character in exciton diffusion.

Energetic disorder is therefore taken into account by the behavior of the Förster radii with temperature. Regarding morphology, spatial disorder may also hinder exciton diffusion. In the context of nanofibers, however, this point is less important since the nanofibers tend to align parallel to each other, leading to highly ordered morphologies. But apart from disorder, recent advances in the techniques involved in the growth of these nanofibers have allowed for the production of extremely thin (~4 Å) layers of these materials^29^, making for an essentially one-dimensional structure for excitons to diffuse. This points to the need of further investigating dimensionality effects in exciton diffusion.

Here, we investigate temperature and dimensionality effects on singlet exciton diffusion in P6P and 6 T. We employ a KMC model that takes experimental radiative lifetimes^29^, the temperature dependent Förster radius (equation 3) and average intermolecular distances^27^ to simulate exciton diffusion in one and two dimensional lattices. The KMC model allows us to understand how the changes in morphology and temperature are translated into diffusion coefficients and diffusion lengths. Results show values for these two parameters to be within typical range for organic materials. We also show that dimensionality affects these features significantly and track this effect to an increase in average intermolecular distance that may come with an increase in the coordination number in the lattice structure. We argue that the distribution of exciton displacements may lead to overestimation in exciton diffusion lengths under experimental conditions. Finally, we investigate the effects on exciton lifetime, diffusion length and emission properties in systems composed of one-dimensional 6 T monolayers alternated with thicker P6P layers for several temperatures.

## Methods

A KMC model was employed to simulate exciton diffusion in P6P and 6 T nanofibers. Two morphologies were employed: one-dimensional and two-dimensional lattices. These simulated, respectively, exciton diffusion between nanofibers in a single layer and in the bulk. In the one-dimensional case, 1000 sites were considered, whereas in the two-dimensional one, a 100 × 100 grid was used. These lattice sizes were chosen for being large enough to prevent border effects.

Excitons were randomly generated throughout the grids. At every time step, the program decides with which neighbor the Förster transfer is going to be attempted. The probability of a neighbor being chosen is proportional to the Förster rate of each transfer. Once the neighbor is chosen, a random number is generated and compared to the probability of Förster transfer to decide whether the exciton hops to a nearby site or recombines. When a hop takes place, the simulation time is increased by an amount given by the inverse Förster rate of the chosen transfer. The time in which recombination takes place is registered along with the total displacement of the exciton during its lifetime. This enables us to obtain the distributions of exciton lifetimes, displacements and speeds. From these data we calculate the corresponding average amounts. Knowledge regarding the position where each exciton recombined also allows for the plotting of emission maps, which show the regions in space where recombination is more common.

To calculate the Förster rates and probabilities, radiative lifetimes, intermolecular distances and Förster radii are required. The latter two were taken from a previous work^27^ that showed the temperature dependence of the P6P and 6 T Förster radii and estimated intermolecular distances in these materials. P6P and 6 T radiative lifetimes were obtained from an earlier experimental work^29^.

In a first round, simulations were run with 100,000 excitons that had their total displacement and lifetimes registered. These simulations ran until all excitons had recombined. In a second round of simulations, 30,000 excitons had their positions registered at each time step up to 2000 ps. The variance of exciton displacements were evaluated as a function of time. These curves were fitted by straight lines, the slopes of which provide the diffusion constants. In all cases, simulations were performed for 8 temperature values ranging from 80 K to 300 K. Exciton-exciton interactions were not taken into account, so our results refer to exciton diffusion in low concentration regimes, as is often the case in experimental setups.

## Results and Discussion

**Figure 1:**
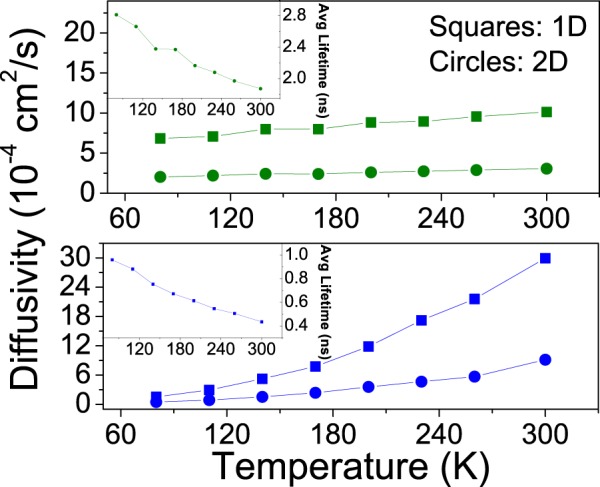
One-dimensional exciton diffusion coefficients as a function of temperature for 6 T (top, green) and P6P (bottom, blue) in the 1D (squares) and 2D (circles) morphologies. Inset: Average exciton lifetimes for different temperatures.

Simulations indicate that singlet excitons are subjected to a normal diffusion process in both morphologies. This is evidenced by the linear relationship between the variance of exciton displacement with time (Fig. 1, SI), which allows us to obtain diffusion coefficients. It is worth noting that even experimental techniques employed to measure diffusion constants and diffusion lengths rely on some form of analytical model for normal diffusion from which these quantities are extracted^6^.

It is important to point out that for the 2D case it is possible to find diffusion coefficients in both *x* and *y* directions. Since both directions are treated equally, these two one-dimensional coefficients are equal. Figure 1-Top shows the one-dimensional diffusion coefficient in 6 T to increase from 6.9 × 10^−4^*cm*^2^/*s* to 10.1 × 10^−4^ *cm*^2^/*s* in the 1D morphology and from 2.0 × 10^−4^ *cm*^2^/*s* to 3.1 × 10^−4^ *cm*^2^/*s* in the 2D one in the temperature range considered. In the case of P6P (Fig. 1-Bottom), the variation of diffusion constant with temperature is larger, ranging from 1.5 × 10^−4^ *cm*^2^/*s* to 30.0 × 10^−4^ *cm*^2^/*s* in the 1D morphology and from 0.4 × 10^−4^ *cm*^2^/*s* to 9.2 × 10^−4^ *cm*^2^/*s* in the 2D one.

These values are within typical values for organic materials^6^. Diffusivity values show an increase with temperature in spite of decreasing average exciton lifetimes (Fig. 1-Insets). This results from the fact that Förster radii either remains constant (in 6 T) or increases with temperature (in P6P). This goes to show that shifts towards lower energies in the absorption and emission spectra are producing increases with temperature in the overlap integral between both spectra, compensating or even outweighing the reductions in lifetime (Fig. 1-Insets).

Furthermore, an increasing detachment between the diffusivities obtained for both morphologies is observed, particularly in P6P. It is clear that 1D diffusion coefficients obtained for 2D morphologies should be inferior than those encountered in 1D morphologies, typically by a factor of two. However, results show this factor to be larger, even though exciton lifetimes are equal and the morphologies are similarly ordered.

**Figure 2:**
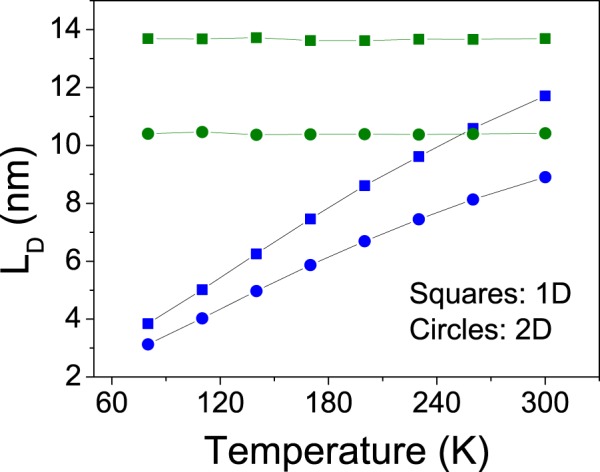
Diffusion lengths in 6 T (green) and P6P (blue) for the 1D (squares) and 2D (circles) morphologies as a function of temperature.

The above mentioned difference also affects exciton diffusion lengths. These may be calculated by means of the equation

4 $${L}_{D}=\sqrt{ZD\tau },$$

where *Z* = 1, 2, 3 is the dimensionality of the process, *D* is the diffusion coefficient and *τ* the average exciton lifetime. In Fig. 2, diffusion lengths for excitons in 6 T (green) and P6P (blue) are shown for the 1D morphology (squares) and for 2D morphologies (circles).

As expected, diffusion length in 6 T remains constant for all temperatures, as its Förster radius does not change. in the case of P6P, however, some features of the diffusion length curves stand out. First, it can be seen that the steep increase in diffusion coefficient observed for the 1D morphology does not translate itself fully to diffusion length. This is the case because of the corresponding large decrease in the average exciton lifetimes, indicating that this characteristic is indeed the main limiting factor to exciton diffusion length as already pointed out in a previous paper^18^. It’s worth mention that all the factors that play a role in diffusion length are already coded in the Förster radius, which makes its temperature dependence the determining factor in the behavior of the curves.

A second important feature is the difference between the total diffusion length calculated for the 1D and 2D morphologies. For P6P, the first one ranges from 3.8 nm to 11.4 nm whereas the latter ranges from 3.1 nm to 8.9 nm. In the case of 6 T, 13.8 nm and 10.4 nm, respectively. Interestingly, the difference is very significant even though the Förster radii, average exciton lifetimes and nanofiber spacing are taken to be the same. To understand this effect, we calculate the distribution of exciton speeds in both morphologies (Fig. 2, SI). The calculations show that, at 300 K, not only average speeds decrease from around 300 *m*/*s* to 200 *m*/*s* in P6P and from 100 *m*/*s* to 65 *m*/*s* in 6 T when going from the 1D to 2D morphologies but there is also an increase in the standard deviation of the distribution.

In the one dimensional case, an exciton has only two neighbors with which a Förster transfer might be attempted. These neighbors are both situated at a 10 Å distance, resulting in equal hopping probabilities and in a single speed for all excitons. When other layers of P6P are juxtaposed, making for a 2D structure in which excitons may hop, the number of neighboring sites becomes 8. Given the square lattice, 4 of the neighbors (vertical and horizontal) are 10 Å away from the exciton position whereas the other 4 (diagonals) are 14.1 Å away. Because of the inverse sixth power dependence of the Förster rate on intermolecular distances (equation 1), this 41% increase in distance results in an 8 fold reduction in the transfer rate ((14.1/10)^6^ ≈ 8). In spite of this reduction, eventually an exciton will attempt a Förster transfer to a farther site. These transfers take a longer time to be performed and will also present a larger probability of recombination when compared to transfers to closer sites.

Therefore, the results indicate that the mere presence of extra adjacent nanofibers whose distances to the exciton site are larger than that of the closer neighbors, but small enough as to still possess non-negligible Förster rates effectively reduces the exciton’s average speed and diffusion length. These results should also apply to molecular crystals, indicating that an increase in coordination number may actually hinder exciton transport.

There are several techniques for measuring exciton diffusion length, the most common being fluorescence quenching in bilayers^6^. This kind of experiment is able to measure one-dimensional diffusion lengths, so it is worth mentioning the one-dimensional projection of the diffusion length calculated for the 2D morphology. This projection was estimated to remain constant at 7.6 nm in 6 T and to range from 2.2 nm to 6.4 nm in P6P, well below the corresponding values obtained for the 1D morphology. Experimental estimates of one-dimensional diffusion length in P6P and 6 T have been obtained using the above mentioned experimental technique^10,28^. The reported values are of 60 nm in 6 T and 30 nm in P6P. These estimations are much higher than the results obtained in our simulations for even the most favorable case, i.e. the 1D morphology.

**Figure 3:**
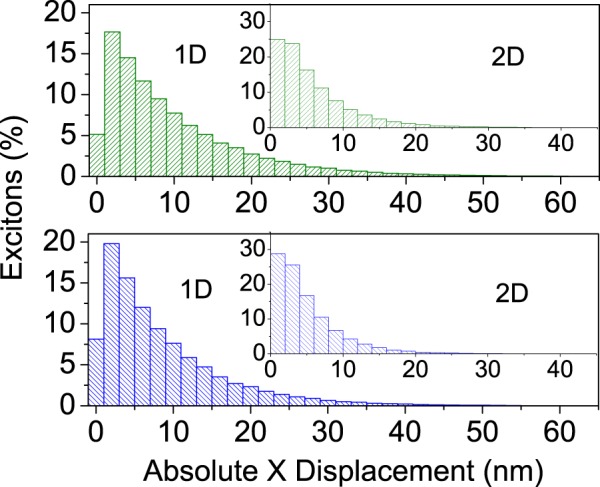
Histograms showing the distribution of absolute displacement in the x axis for excitons at 300 K in 6 T (top) and P6P (bottom) for 1D and 2D (inset) morphologies.

Given the observed mismatch between the experimental estimation and the simulations, it is worth considering the distribution of one-dimensional absolute displacements for excitons in both morphologies. Figure 3 shows these distributions at 300 K. It can be seen that absolute displacements of around 60 nm in 6 T and 30 nm in P6P correspond to the tail of the distributions. Only 3% and 0.1% of excitons recombine at least 30 nm away form their original positions in the 1D and 2D morphologies, respectively. In the case of 6 T, for a 60 nm distance, these numbers drop to 0.2% and 0.002%, respectively. Importantly, every exciton has a similar probability of recombination regardless of its diffusion length. By chance, some of them live longer than the others, which allows them to reach larger distances. It is also worth noting that in the simulations excitons that may have reached the 30 nm or 60 nm mark could have diffused back, resulting in lower values than would have been observed experimentally, where exciton quenching prevents this return. However, this fact is not enough to explain the large difference between experimental and simulated values. We conclude that these experimental values overestimate singlet exciton diffusion length in both 6 T and P6P–which is by definition an average value–rather pointing to their maximum one-dimensional displacement. It is not to say that there is necessarily a discrepancy between simulation and experiment. It is just that the experiment seems to be measuring the distances covered by a small number of excitons and taking these values to be representative of the average distance an exciton travels.

**Figure 4:**
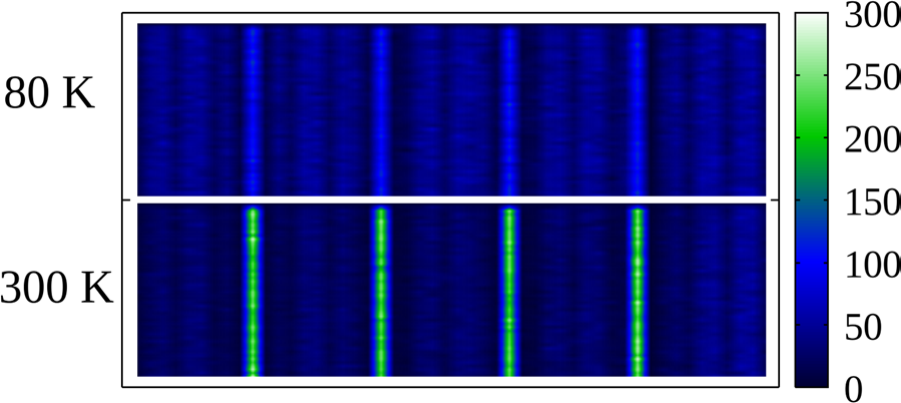
Emission map for singlet excitons in a P6P/6 T alternate layered morphology at 80 K (top) and 300 K (bottom).

Finally, we turn to the effects of combining P6P and 6 T nanofibers in a morphology that alternates 6 T monolayers (~4 Å thick) with thicker P6P layers (~160 Å). This setup has been experimentally investigated^10,29^ and holds interest for at least two reasons. First, the combination of these two materials results in interesting exciton dynamics. Because of their spectroscopic properties, Förster transfers are possible from P6P to 6 T. Experimental estimates put the Förster radius of this transfer around 36 Å^10^. However, the reversed transfer, from 6 T to P6P, is not allowed^27^. As shown in Fig. 4, which presents the simulated emission maps for excitons in the alternate layered morphology, this results in changes in the emitted color from mainly blue to green as temperature increases. This is the case because diffusion lengths in P6P increase, allowing excitons to reach the interface and migrate towards the 6 T monolayers, changing the proportion of excitons that recombine in both materials.

**Figure 5:**
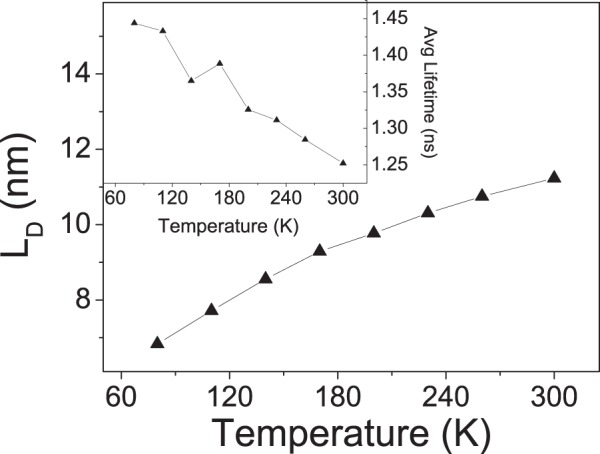
Exciton diffusion lengths in a system composed of alternating layers of P6P and 6 T as a function of temperature. (Inset) Average exciton lifetimes in this system.

A second reason of interest in such a system is the combination of two-dimensional–P6P layers–and one-dimensional–6 T monolayers–structures for excitons to diffuse. The effects of temperature on exciton diffusion length and lifetime are shown in Fig. 5. It can be seen that exciton lifetimes decrease from 1.4 ns to 1.3 ns in the 80 K to 300 K range. In this same range, the diffusion length increases from 6.8 nm to 11.2 nm. Interestingly, we note that the average lifetimes present a temperature sensitivity similar to the one in pure 6 T, changing only mildly in the temperature range considered. The reason is that exciton lifetimes are longer in 6 T than in P6P, as shown in the Fig. 1. When an exciton hops from P6P to 6 T, it ends up living longer, pushing average lifetimes as a whole upwards. Meanwhile, diffusion lengths follow the trend seen for pure P6P, with a temperature activated profile. This is expected since in the model excitons are generated in the P6P layers, which are much larger than the 6 T ones.

## Conclusions

In summary, we investigate temperature and dimensionality effects on exciton diffusion in 6 T and P6P nanofibers. Both materials present different temperature sensitivities, with P6P presenting a temperature activated behavior whereas 6 T shows virtually no temperature dependence. As to dimensionality effects, our results show that exciton diffusion coefficients and diffusion lengths may be considerably affected by the coordination number found in the morphology of the system, particularly if a change in this number actually corresponds to an increase in the average intermolecular distance. We observe that because of this effect, simulations performed in 1D morphologies showed much larger diffusion coefficients and diffusion lengths than their 2D counterparts in spite of otherwise completely equal simulation parameters. We find further that in spite of considerable gains in diffusivity with temperature, diffusion lengths do not follow a similar increase, being offset by decreasing average exciton lifetimes.

We calculate one-dimensional exciton diffusion length in P6P to be around 6 nm at room temperature with a corresponding diffusion constant of 9 × 10^−4^ *cm*^2^/*s*. In 6 T monolayers, these parameters are estimated to be 13.7 nm and 10 × 10^−4^ *cm*^2^/*s*. These values are within typical values for organic materials. However, comparison between the distribution of exciton displacements with experimental estimates of diffusion length in both 6 T and P6P suggests that the experimental technique estimates maximum exciton displacement rather than diffusion lengths, which are an average quantity. This may result in large overestimation of exciton diffusion lengths. It is also worth noting that exciton-exciton interactions were not taken into account. Since such effects tend to reduce diffusion lengths, our estimates can be seen as upper limits. Thus, the inclusion of these effects in the model would not modify the conclusions we obtained for this paper.

Finally, we show the effects of combining P6P and 6 T in an alternate layered morphology. Emission maps show the change in emitted color with temperature caused by the corresponding increase in exciton diffusion lengths reaching around 11 nm. At the same time, average exciton lifetimes become less sensitive to temperature effects staying around the 1 ns mark. Our findings provide a better physical insight into the factors that play a role in exciton diffusion in general and in the particular case of P6P and 6 T nanofibers. More importantly, they also point to the necessity of reevaluating experimental estimates of exciton diffusion length.

## Electronic supplementary material

Supplementary Information
