# Supplementary material for: Exciton Diffusion in Organic Nanofibers: A Monte Carlo Study on the Effects of Temperature and Dimensionality
Source: Sci Rep. 2018 Sep 19;8:14066. doi: 10.1038/s41598-018-32232-5 (PMC6145872; doi:10.1038/s41598-018-32232-5)
Supplement: Supplementary file 1 — Supplementary Information [file 41598_2018_32232_MOESM1_ESM.pdf]

Exciton Diffusion in Organic Nanofibers: A Monte Carlo Study on the Effects of Temperature and Dimensionality

Leonardo Evaristo de Sousa[1], Demétrio Antônio da Silva Filho[1,*], Rafael Timóteo de Sousa Jr.[2], and Pedro Henrique de Oliveira Neto[1]

[1]Institute of Physics, University of Brasília, 70.919-970 Brasília, Brazil
[2]Department of Electrical Engineering, University of Brasília, 70.919-970 Brasília, Brazil
[*]dasf@unb.br

Supplementary Information

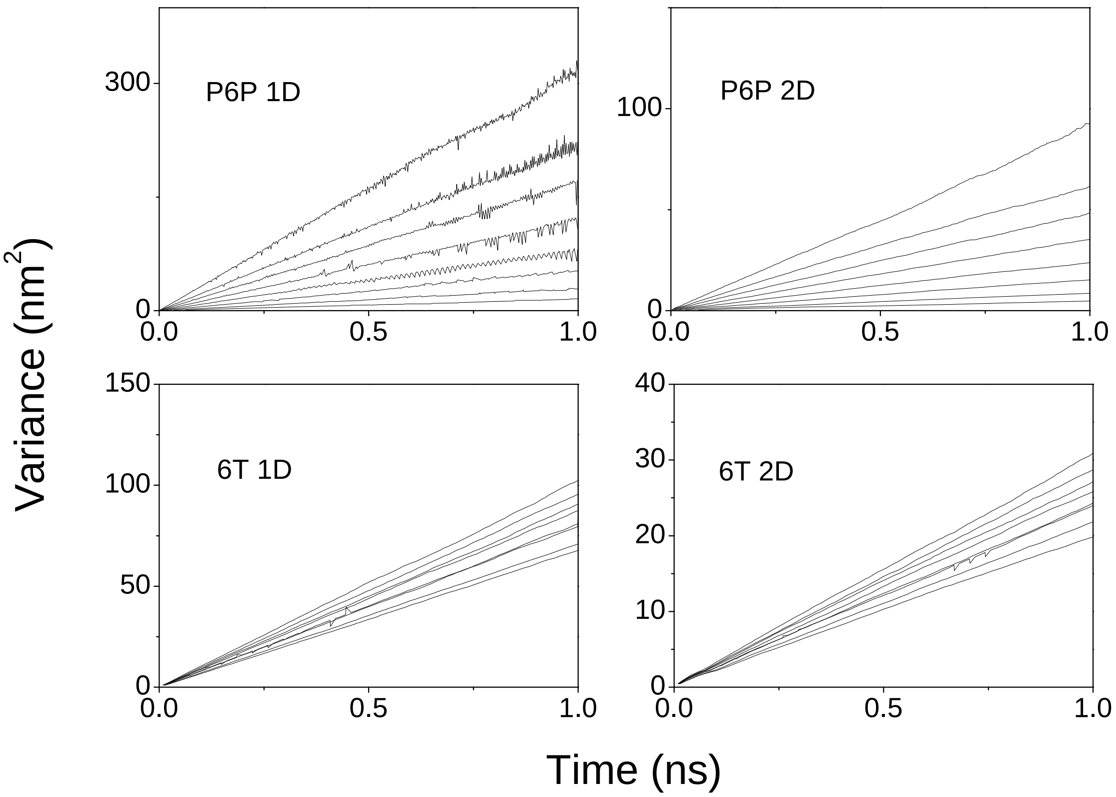

Figure 1. Variance of exciton displacements as a function of time for different materials and dimensions. The 2D results refer to the variance of the x coordinate. Similar results are found for the y coordinate. All curves show a linear behavior, typical of normal diffusion.

Figure 1 shows the time evolution of the variance of exciton displacements for both P6P and 6T in their one and two-dimensional morphologies. Each curve represents a simulation performed at a certain temperature. It can be seen that such curves may be well described by straight lines. This is a behavior that characterizes normal diffusion. To confirm this, all curves were fitted with a function $y = At^{\alpha}$. In all cases, the α values were found to be higher than 0.9, confirming that the linearity consideration is appropriate. To obtain diffusion constants, the slopes of the straight lines that fit these curves are extracted. This is how the values shown in Figure 1 of the main article were found.

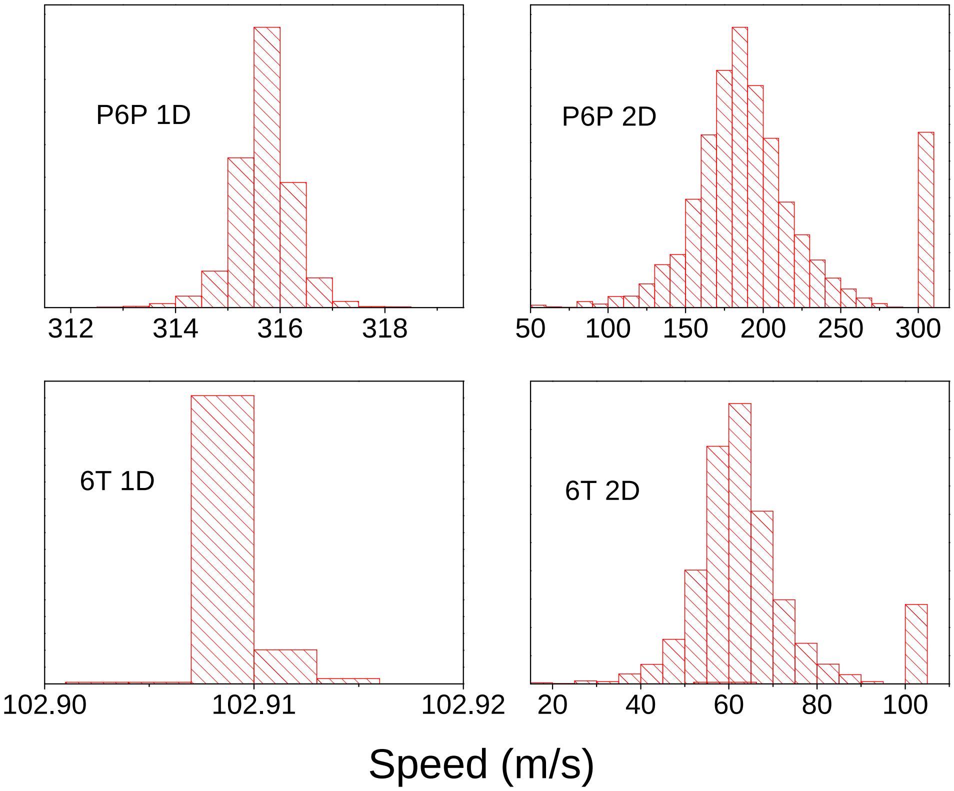

Figure 2. Speed distributions for excitons in P6P and 6T at 300 K.

Figure 2 presents the distributions of exciton speeds for P6P and 6T in both one and two-dimensional morphologies. The KMC program records the total distance traveled by each exciton as well as its lifetime. From these two informations we calculate the speed of each exciton in the simulation. These are, in turn, plotted as the histograms showed n Figure 2. It can be seen that for one-dimensional morphologies the distributions are very narrow. As explained in the main text, this is the result of the existence of only two equally distant neighbors. For the two-dimensional morphologies, the speed distributions are much broader, given that excitons may hop in different directions. It is worth noting that these two-dimensional distributions present a large peak at the 300 m/s and 100 m/s mark for P6P and 6T, respectively. These peaks account for the excitons that during their lifetime moved only in the directions of minimal hopping distances, which are also the directions for which Förster rates and probabilities are maximal. This is evidenced by the fact that speed values at these peak correspond to the speed values shown in the one-dimensional case for both materials.
